# Hollow Mesoporous Microspheres Coating for Super-Hydrophobicity Wood with High Thermostability and Abrasion Performance

**DOI:** 10.3390/polym12122856

**Published:** 2020-11-29

**Authors:** Rui Yang, Shida Zuo, Beibei Song, Haiyan Mao, Zhenhua Huang, Yingji Wu, Liping Cai, Shengbo Ge, Hailan Lian, Changlei Xia

**Affiliations:** 1 Co-Innovation Center of Efficient Processing and Utilization of Forest Resources, College of Materials Science and Engineering, Nanjing Forestry University, Nanjing 210037, China; yangrui@njfu.edu.cn (R.Y.); zuoshida@njfu.edu.cn (S.Z.); song_beibei@163.com (B.S.); wuyingji@njfu.edu.cn (Y.W.); geshengbo@njfu.edu.cn (S.G.); lianhailan@njfu.edu.cn (H.L.); 2 Dehua Tubaobao New Decoration Material Co., Ltd., Huzhou 313200, China; 3 Department of Chemical and Biomolecular Engineering, University of California, Berkeley, CA 94720, USA; 4 Jiangsu Chenguang Coating Co., Ltd., Changzhou 213164, China; 5 Department of Mechanical Engineering, University of North Texas, Denton, TX 76207, USA; zhenhua.huang@unt.edu (Z.H.); liping.cai@unt.edu (L.C.)

**Keywords:** wood, hydrophobic, hollow mesoporous, microsphere

## Abstract

Super-hydrophobic phenomena generally exist in nature, and wood can also obtain hydrophobicity by specific processing on the surface, being like the construction of microscale rough surface or decoration with low surface energy materials. In this research, the formation of hydrophobic layers on wood surface was investigated without breaking the wood’s original structure. The core-shell structure particles were prepared by penetrating orthosilicate and polystyrene into the hollow mesoporous microsphere structure with tetrahydrofuran. A wood sample was coated with polydimethylsiloxane (PDMS) resin layer to enhance the adhesion of nano and micron hollow mesoporous microsphere on its surface. According to the surface structure of super-hydrophobic subjects in nature, the nano and micron hollow mesoporous microsphere were sprayed with different ratios several times to form a hydrophobic surface. The water contact angle could reach 150°, revealing that the hydrophobic behavior of the nano and micron hollow mesoporous microsphere coating was achieved. The microstructures of wood samples were examined by the scanning electron microscopy, and the chemical functional groups were investigated by the Fourier transform infrared; both verified that the hydrophobic surface was successfully coated. The thermogravimetric examination revealed the improved thermal stability of the hydrophobic wood. The scratch test was used to measure the abrasion resistance of the nano and micron hollow mesoporous microsphere coatings on wood surface. It was suggested that the nano and micron hollow mesoporous microsphere coating was an effective method to fabricate extremely hydrophobic wood products.

## 1. Introduction

Wood is a non-polluting, resource-rich, natural, and renewable biomass material. It has been placed in an extremely important position in various industries such as furniture, construction, ships, musical instruments, handicrafts, and buildings [1,2,3]. People have been looking for ways to make better use of wood, but the natural characteristics of wood materials limit the further use of wood [4]. Wood exhibits extremely strong moisture absorption due to the abundant hydroxyl groups [5,6]. The change of moisture in wood will inevitably lead to the change of wood size and anti-corrosion capability [7,8,9]. These uncertain factors will affect the scope of application and service life of wood [10,11].

The bionic construction of super-hydrophobic wood could not only improve the hydrophobicity of the wood, but also effectively improve the self-cleaning type of the wood surface, which avoids a series of defects such as cracking, deformation, decay, mildew, and discoloration caused by water absorption. It can provide wood with good performances of microwave absorption, magnetism, electrical conductivity, flame retardancy, and other functions, which has important research value and practical significance for the utilization of high value-added wood [12,13,14,15,16]. The super-hydrophobic bionic surface construction of wood has gradually become one of the main research hotspots in the functional modification of wood [13].

To enhance the hydrophobicity of wood, a lot of modification studies have been conducted. Methods to improve hydrophobicity can be summarized into two types, such as improving the surface roughness of wood and using low surface energy materials [17]. Methods of constructing microscopic roughness on solid surfaces include the electrospinning, template method, layer-by-layer self-assembly, sol-gel, anodic oxidation, chemical vapor deposition, chemical etching, and electrochemical deposition [18,19,20,21,22,23,24,25]. However, due to the special properties of wood, such as uneven microstructure and uneven physical characteristics, the electrospinning and other methods are suitable for constructing continuous fiber super-hydrophobic surfaces, which are widely used in fabrics [26]. The methods are suitable for creating wood superhydrophobic surface roughness including surface coating, wet chemical method, hydrothermal method, layer-by-layer self-assembly, and sol-gel method [27,28].

When preparing superhydrophobic surfaces, it is easier to reduce the free energy of the material surface at the technical level [29]. Therefore, the key is to construct a suitable micro-nano rough structure. Yang et al. used low-temperature chemical vapor deposition technology to vacuumize the experimental device and inject water vapor and dimethyldichlorosilane gas to prepare polydimethylsiloxane (PDMS)-coated wood with a hydrophobic function. The contact angle (CA) is up to 157° [30]. However, the vapor deposition method can construct a fine and orderly array structure on the substrate surface. However, a closed reaction vessel is required, the reaction conditions are harsh, and the growth rate is slow, which takes a long time.

In this paper, starting from the superhydrophobic phenomenon in nature, on the basis of existing related technology, by using tetraethyl orthosilicate (TEOS) to produce silica particles, the superhydrophobic surface of wood is constructed, thereby expanding the scope of wood use and generating better economic benefits. The core-shell structure particles are prepared using tetraethyl orthosilicate and polystyrene, and the particles are processed into a hollow mesoporous structure through the tetrahydrofuran (THF) treatment.

The purpose of this research is to use Chinese fir as a raw material to develop a hydrophobic coating with micro-nano structure on its surface. To keep the original structure of the wood surface unchanged, it is necessary to spray a layer of resin substrate treatment on the surface of the sample to enhance the adhesion of the micro-nano particles to the wood. The core-shell structure particles are prepared using tetraethyl orthosilicate and polystyrene, and the particles are processed into a hollow mesoporous structure through the THF treatment. After that, the produced nano and micro hollow mesoporous microspheres are coated on the wood surface in different proportions to build a bionic hydrophobic structure.

## 2. Materials and Methods

### 2.1. Preparation Micro/Nano Hollow Mesoporous Silica

#### 2.1.1. Preparation of Nano Polystyrene

**Figure 1:**
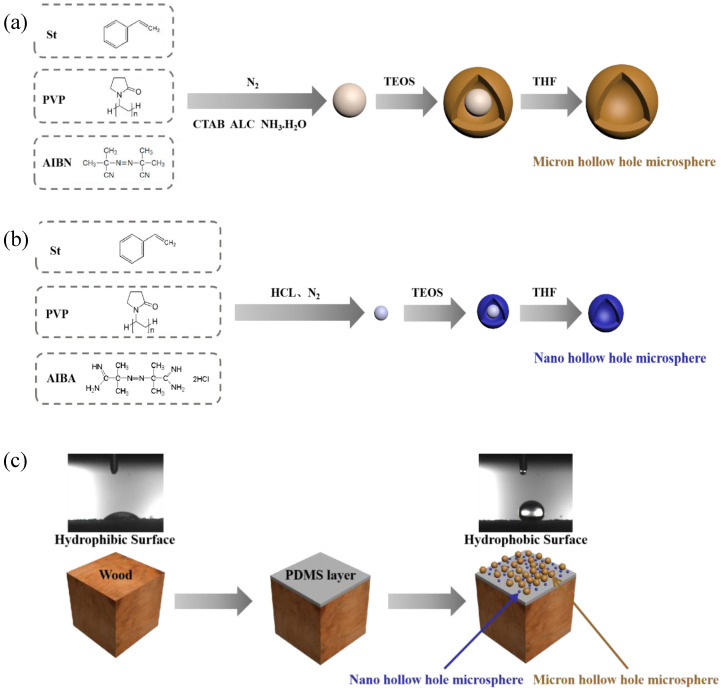
Schematic illustration of preparation process. (**a**) Construction of micron hollow hole microsphere; (**b**) construction of nano hollow hole microsphere; (**c**) description of superhydrophobic surface formation on wood.

Emulsion polymerization was used to prepare nano-scale polystyrene particles. Styrene (St) and polyvinylpyrrolidone (PVP) with a mass ratio of 1:3 was added to a three-necked flask (250 mL), some deionized water was also added, and the mixture was stirred at 120 rpm for 10 min at room temperature. Then azobisisobutyronitrile (AIBN) was dissolved in water at a ratio of 1:25 and added to the above mixed solution. Finally, the mixed solution was deoxidized by passing N_2_ at room temperature for 1 h, heated to 75 °C, and reacted at 120 rpm for 24 h to obtain a colloidal solution (Figure 1b).

#### 2.1.2. Preparation of Micron Polystyrene

Dispersion polymerization was used to prepare micron-sized polystyrene particles. St, PVP, absolute ethanol, and deionized water with a mass ratio of 40:3:160:10 were added to a three-necked flask (250 mL). The mixed solution was stirred at 120 rpm for 10 min at room temperature. Then a little AIBN was added to the mixed solution. After being deoxygenated by passing N_2_ at room temperature for 1 h, the mixture was heated to 75 °C, and reacted at 120 rpm for 24 h to finally obtain a microsphere solution. The microspheres were washed twice with deionized water and absolute ethanol by centrifugation, and finally dispersed in ethanol solution with a solid content of 0.7% in the absolute ethanol solution (Figure 1a).

#### 2.1.3. Nano Core-Shell Structure Polystyrene/Silica Microspheres

Some nanoscale sols prepared in Section 2.1.1 were adjusted to pH 4.0 with HCl solution, and then TEOS was added dropwise at a mass ratio of 1:20. With the hydrolysis and condensation of TEOS at 40 °C for 24 h, the dispersion of core-shell structured nanoparticles was prepared. The obtained core-shell particles were centrifuged and washed twice with a large amount of water and ethanol, and finally an ethanol dispersion of nano-scale particles was obtained (Figure 1b).

#### 2.1.4. Micron Core-Shell Structure Polystyrene/Silica Microspheres

The micron-sized microsphere solution prepared in Section 2.1.2, and the cetyltrimethylammonium chloride (CTAB), ammonia, deionized water and absolute ethanol mixture with a ratio of 10:1:2:100:50 were added into a three-necked flask (250 mL). TEOS with a mass ratio of 3:20 was then added dropwise to the microsphere solution. The silicon source TEOS was hydrolyzed and condensed at 120 rpm and 40 °C for 24 h to obtain the final core-shell microsphere product. The obtained core-shell particles were centrifuged and washed twice with a large amount of water and ethanol solution to finally obtain an ethanol dispersion of micron-sized particles (Figure 1a).

#### 2.1.5. Preparation of Nano Hollow Mesoporous Silica

The ethanol solution of nanoparticles prepared in Section 2.1.1 was centrifuged, and the core-shell particles were eluted into a 250 mL flask by a large amount of THF solution. The mixture was stirred at 55 °C at 250 rpm for 24 h to obtain a translucent mixture. The obtained particles were washed three times with THF circular centrifugation and finally dispersed in ethanol solution and dried for later use (Figure 1b).

#### 2.1.6. Preparation of Micron Hollow Mesoporous Silica

The ethanol solution of micron-sized nanoparticles in Section 2.1.3 was centrifuged, and the core-shell particles were eluted into a 250 mL flask with a large amount of THF solution. The mixture was stirred at 55 °C at 250 rpm for 24 h to obtain a half transparent mixture. The obtained particles were washed three times with THF circular centrifugation and finally dispersed in ethanol solution and dried for later use (Figure 1a).

### 2.2. Preparation of Superhydrophobic Surface

Chinese Fir wood obtained from Jiaozuo Forest Company (Nanjing, China), used as raw material, was cut into specimens with the size of 10 mm × 10 mm × 10 mm (L × T × R). The density was 0.41 g/cm^3^. Epoxy resin (diglycidyl ether of bisphenol A) and polyamide resin were dissolved in THF at a ratio of 3:2:600 to prepare a basic epoxy resin solution. PDMS precursor and TEOS were dissolved in n-heptane at a mass ratio of 25:3:250 and stirred for 1 h, then a trace amount of dibutyltin dilaurate was added and stirred for 5 min to obtain PDMS solution. The slides were washed continuously in acetone, deionized water and ethanol under ultrasonic conditions, and the fir was baked at 100 °C to be dried absolutely for late use. The wood cross section and tangential section surface was sprayed with epoxy resin base solution and cured at 80 °C for 10 min. Then, the ethanol dispersion of core-shell particles with a mass fraction of 0.7 wt% (spraying experiments with a mass fraction of 0.4 wt% and 1.0 wt%) were sprayed using spray gun (F-2, Rongpeng Pneumatic Tools Co. LTD., Zhejiang, China) onto the epoxy-treated wood surface at room temperature. With the rapid evaporation of ethanol, the core-shell particles were deposited on the cross section and tangential section surface of the substrate. The spraying process was repeated 15 times (5, 10, and 20 spray assembly processes were also tested). The coating was then baked at 100 °C for 1 h. Finally, the core-shell structure coating was sprayed with PDMS solution for 10 s, and the final coating was baked at 100 °C for 2 h to obtain the final stable superhydrophobic surface (Figure 1c).

### 2.3. Superhydrophobicity Measurement

The coated glass sheet was placed on the CA tester (JC2000C POWEREACH, Shanghai, China) to measure the static CA of the coating surface. The diameter section of the Chinese fir specimen was the test surface. In the experiment, three samples were selected under each condition, and three points were selected on each sample, respectively placed at the center point of the radial section and two edge points. The static CA was measured 100 s after a water droplet (5 µL) dropped on the wood surface. The test started from the drop of water on the surface, and the test was performed every 10s for a total of 100 s. There was a total of 11 values; the final value was the average of the nine points tested on each specimen.

### 2.4. Surface Observation

#### 2.4.1. Core-Shell Particle Surface

The produced polystyrene, core-shell structured nanoparticles, mesoporous nanoparticles, and micron/nanoparticles were dropped on the silicon wafer to make samples. The solid concentration should not be too large to prevent unchecked results in the detection. The silicon wafer covered with the sample was glued on the stage. After spraying gold, it was placed into the Quanta 200 field emission scanning electron microscope (Quanta 200 SEM, Hillsboro, OR, USA) for observation, and the comparison pictures of three samples under different magnification were taken.

#### 2.4.2. Wood Surface

The untreated wood sample was selected and sprayed with resin substrate. The specimens with the best hydrophobic conditions were ready for testing. With a blade, the wooden block was cut into a test piece of 5 mm × 2 mm × 2 mm, and the top was smoothed with a glass knife, then processed, and glued on the stage. After that, the sample was placed into the ion sputtering instrument (SCD005) and vacuum (pump to 10^−1^ Pa) for gold spraying (current 15 mA, 6 s), and then was transferred into the TM3000 scanning electron microscope (TM3000, HITACHI, Tokyo, Japan) for observation, in which three charts from different tests were taken for the comparison.

### 2.5. FT-IR Analysis

The infrared spectrometer (Nicolet380, FT-IR, Watha, MA, USA) was used to analyze the cross-section of samples. The measuring range was 500–4000cm^−1^, resolution was 4 cm^−1^, scanning times was 64 times, and the accessories was Smart Specul ATR Accessory. The ATR horizontal attenuation total reflection accessory was installed on the FT-IR spectrometer, the surface was wiped with absolute ethanol before each test, and the background single-channel spectrum was pre-measured before the test. The samples were placed above the ATR accessory detector and pulled. The pressing rod was compacted, and the infrared spectrum of each test piece was measured with the OMNIC software and then analyzed.

### 2.6. XRD Analysis

Since the originally processed specimen met the requirements of the machine test, no additional processing was performed on the specimen. The test piece was clamped on the stage to ensure that the surface to be measured was parallel to the stage. The samples were scanned by the Discovery Diffractometer (Ultima IV, Rigaku, Japan) from 2θ = 10° to 80°. The Segal peak height method was utilized to calculate the relative crystalline values (CrI) [31].

### 2.7. TG Experiments

The thermal properties of hydrophobic wood and untreated wood were measured by the thermogravimetric (TG, TGA55 of TA Instruments, New Castle, DE, USA) analysis. The initial weight of TG sample for untreated wood, PDMS resin layer on wood and superhydrophobic surface with nano/micro hollow mesoporous microsphere layer on wood was maintained at around 7 to 9 mg. All samples for TG test were ground into around 100 meshes. The analysis was conducted in the nitrogen flow with the temperature from 20 to 800 °C at a rate of 5 °C/min. Triplicate was completed for each measurement, and the average weight loss curves were shown.

### 2.8. Mechanical Durability of the Hydrophobic Coatings

To evaluate the mechanical stability of the hydrophobic surface of wood with different sections, abrasion tests were carried out using the sandpapers (Alibaba, Hangzhou, China) with 1500 mesh [32]. During the test, the sandpaper was moved on the superhydrophobic surface of wood with close contact and kept in one direction with a constant speed. The external force was 100 g weight pressure. Ten cycles of resistance tests were performed to evaluate the serious condition during use. After that, the water contact angles (CAs) of the hydrophobic surface were measured by the Theta Optical CA Tester (JC2000C POWEREACH, Shanghai, China) again.

## 3. Results and Discussion

### 3.1. Microtopography of Hollow Mesoporous Microspheres and Wood Surface

**Figure 2:**
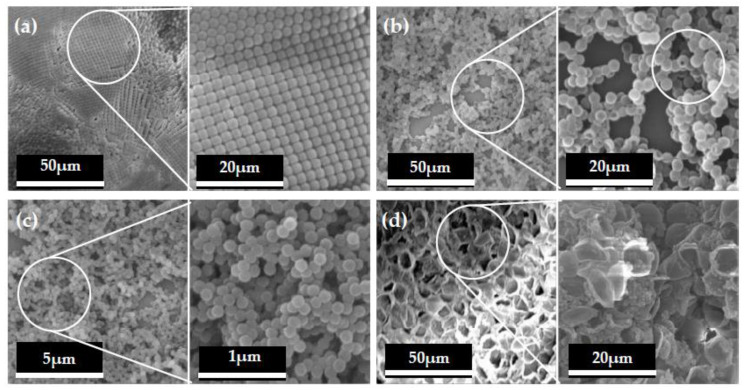
SEM morphology photos of micro-spherical appearance. (**a**) Polystyrene; (**b**) micron hollow mesoporous microspheres; (**c**) nano hollow mesoporous microspheres; (**d**) nano/Micro hollow mesoporous microspheres.

Figure 2a shows the polystyrene particles produced in Section 2.1.1 and Section 2.1.2. It could be seen that the particle size of polystyrene was within 100 nm, which proved that it reached the nanometer level. Meanwhile, the micron dimension of the micro hollow mesoporous microspheres can also be seen in Figure 2b, which showed that the micro hollow mesoporous microspheres were successfully produced for construction of a hydrophobic surface. It can also be seen from the marked circle in Figure 2b that the core-shell hollow structures were achieved obviously in micro hollow mesoporous microspheres. At the same time, Figure 2c shows the dimension of the nano hollow mesoporous microspheres. It was illustrated that the nano hollow mesoporous microspheres were almost smaller than 100 nm and maintained preferable microsphere morphology. Based on Figure 2d, it was revealed that the nano and micro hollow mesoporous microspheres were mixed to achieve the required superhydrophobic surface on wood using the proposed methods in this research. In addition, the nano and micro hollow mesoporous microspheres were homodisperse for increasing the roughness of surface.

**Figure 3:**
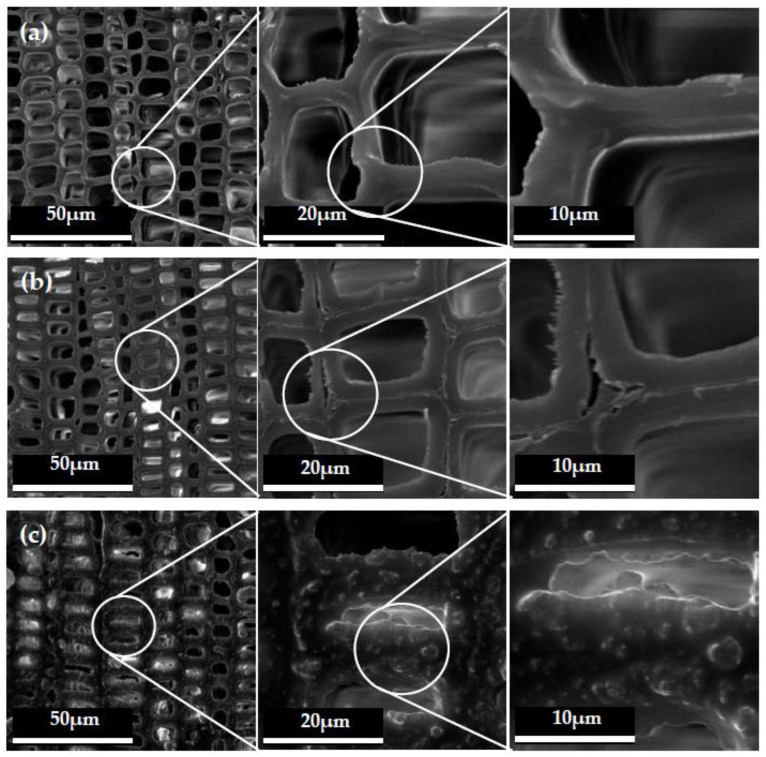
SEM morphology of wood cross section; (**a**) untreated wood; (**b**) with PDMS resin layer; (**c**) the hydrophobic surface with nano and micro hollow mesoporous microspheres.

The SEM morphologies with 600, 2500 and 5000 magnification times of the untreated fir wood with cross sections are shown in Figure 3a. It could be clearly seen that the surface of the untreated wood was very smooth, and the cell walls were clearly visible. The SEM morphologies with different magnification times of the cross section with PDMS layer of the treated wood are also shown in Figure 3b. It was observed that the wood cell wall surface still maintained the original microstructure at 600 magnification times, but when the magnification times reached 5000, the imperceptible changes could be seen. Between the cell walls and in the cell cavity, some “burrs” could be clearly observed. These “burrs” were formed by the curing of the resin base liquid sprayed on the surface of the wood. Figure 3c presents the surface with nano and micro hollow mesoporous microspheres. It was illustrated that there were obviously micron and nano particles on the wood cell walls, which uniformly distributed on the wood surface. It was revealed that the nano and micro hollow mesoporous microspheres successfully and homogeneously attached on the wood surface, which endowed wood surfaces with hydrophobic character.

**Figure 4:**
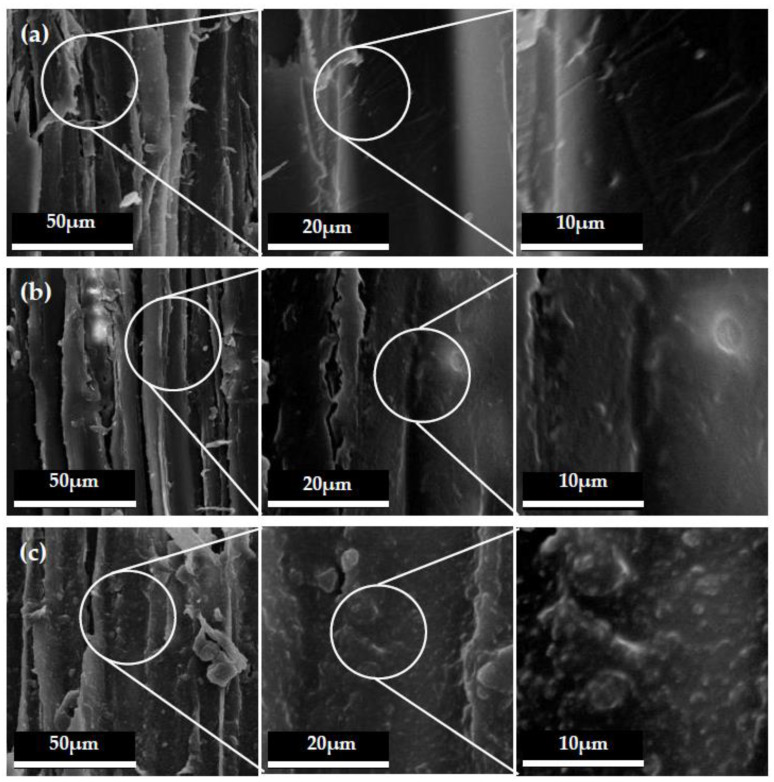
SEM morphology of wood tangential section: (**a**) untreated wood, (**b**) with PDMS resin layer, (**c**) the hydrophobic surface with nano and micro hollow mesoporous microspheres.

The microscopic topography with different magnification times of the tangential section of the wood are shown in Figure 4. As seen in Figure 4a, the inner cell walls of the untreated wood were clear and smooth. After PDMS was coated on the wood, the morphology of wood cell walls was covered with a thin film of resin layer, as shown in Figure 4b. Moreover, the mixed micro-nano hollow mesoporous microspheres coating on wood surface was also clearly seen in Figure 4c on the tangential section. This was the reason why the hydrophobic performance was greatly improved. Combining Figure 3 and Figure 4, it was revealed that the micro-nano mixed hollow mesoporous microspheres did not change the structure of the wood itself, but a layer of specific structure was added to the original structure of the wood. When the appropriate proportion of mixed micro-nano hollow mesoporous microspheres and the suitable number of spraying times were chosen, the hydrophobic surface on wood would successfully be constructed.

### 3.2. Superhydrophobicity of Wood Surface

**Figure 5:**
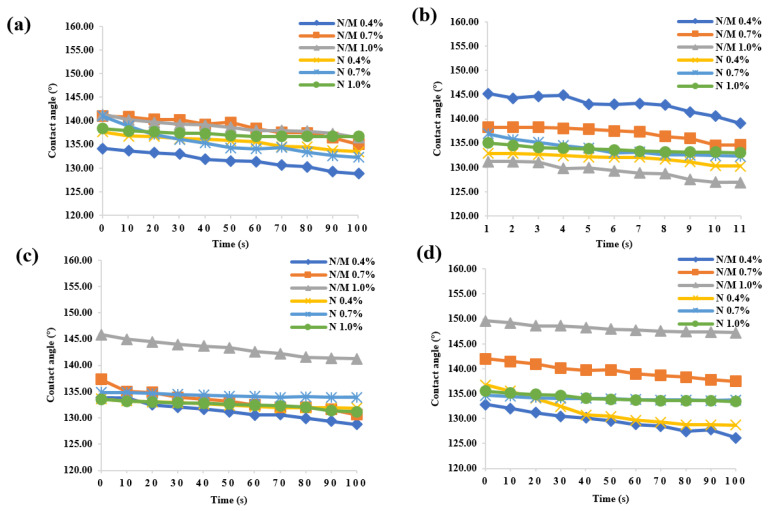
The water contact angles of wood samples with different treatment conditions. (**a**) Spraying layers 5 times, (**b**) spraying layers 10 times, (**c**) spraying layers 15 times and (**d**) spraying layers 20 times.

Figure 5 shows the water CAs of wood samples under different treatment conditions with mixed nano and micro hollow mesoporous microspheres. The water CAs greater than 90° denote hydrophobic surfaces (non-wettable), while those smaller than 90° represent hydrophilic ones. It was obviously seen that all the wood samples treated in this research achieved hydrophobic surface, and the hydrophobic effect also maintained very well as time went by. The hydrophobic effect was different with different treatment conditions. When the concentration of nano and micron mixed hollow mesoporous microspheres was 0.4% and the spraying number was 10 times, the hydrophobic effect was obvious and the maximum water CA was 145°, which realized effective hydrophobic impact. Moreover, when the concentration of mixed particles was 0.7% and the treatment condition were 20 times, the hydrophobic effect was the best, and the CA was up to 142°. At the same time, when the concentration of nano and micron mixed hollow mesoporous microspheres was 0.7% and the spraying number was five times, the hydrophobic effect was the best, and the maximum CA was 141°. Under all treatment conditions, when the concentration of mixed particles was 1.0% and the treatment conditions were 20 times, the hydrophobic effect was the best, achieving the highest CA of 150°, i.e., super-hydrophobic property. With the increase of coating number, the contact angle of wood surface was larger, which indicated that the stacking of nano and micron mixed hollow mesoporous microspheres spraying layers can improve the hydrophobicity of wood surface. At the same time, considering the manufacturing cost, the suitable concentration ratio was beneficial to enhance the hydrophobicity of wood. The optimum concentration was conducive to the more effective and uniform dispersion of the nano and micron mixed hollow mesoporous microspheres, increasing the roughness of the wood surface and constructing the hydrophobic surface. In addition, the water CAs all remained hydrophobic after water dripped onto the wood surface after 100 s, which meant outstanding hydrophobic stability.

**Figure 6:**
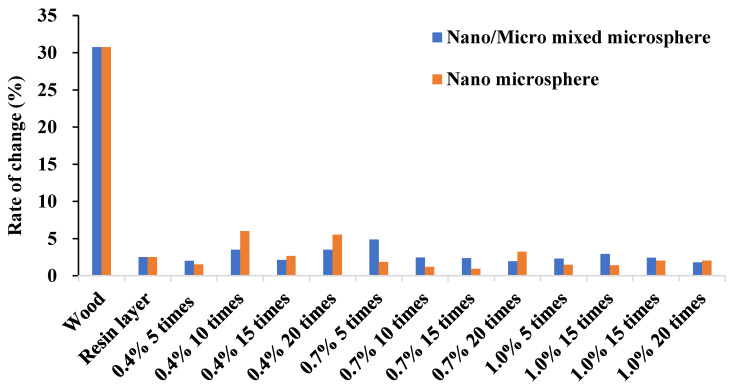
The rate of change of contact angle with different treating conditions.

The change rate of CA under different treatment conditions is shown in Figure 6. It could be seen that after coating layers on the wood surface, the change rate of CA of wood sample became significantly lower. As shown in Figure 6, the CA change rate of the wood surface treated with PDMS resin and nano and micro hollow mesoporous microspheres was small, indicating that the surface of the treated wood had better hydrophobic properties and excellent durability. After water dropped on the wood surface for 100 s, the morphology of the hydrophobic surface of the wood did not change significantly, and the change rate was below 0.06. Figure 6 also shows that, when the concentration of microspheres gradually increased, the CA change rate tended to be stable and the balance was about 2.5%, and the hydrophobic persistence of the wood surface tended to be stable. It was obvious that when the concentration of microspheres in the treatment was low, the CA change rate of pure nano-microspheres-treated wood was high. Meanwhile, the durability of the wood surface treated by the mixed microspheres was improved, and when the concentration of the microspheres increased, the treatment of pure nanospheres had better durability of the hydrophobic properties of the wood surface compared with the treatment of the mixed microspheres. In the pure nanoparticle treatment, the treatment condition was a concentration of 0.7%. When spraying 15 times, the hydrophobic durability was the best, and the change rate was only 0.9%. In addition, in the mixed particles, when the concentration was 1.0% and spraying 20 times, the highest hydrophobic effect was presented, and the rate of change was 1.8%.

### 3.3. Chemical Structure Investigation of Wood

**Figure 7:**
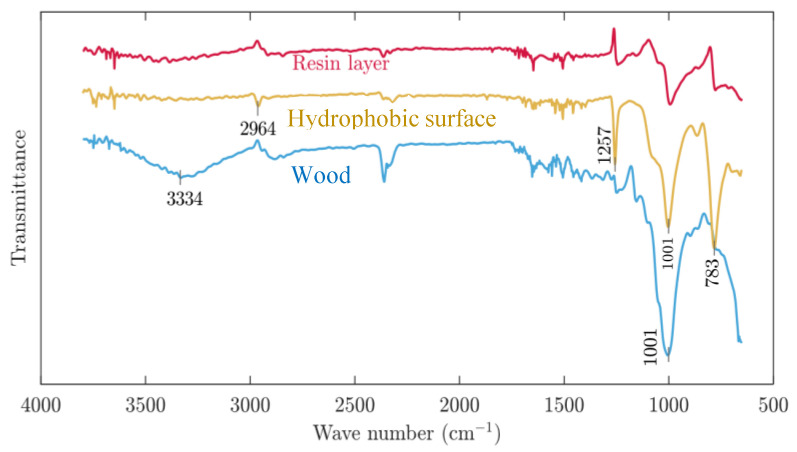
The infrared spectrum of the wood surface before and after treatment.

The FT-IR examination used to explore the changes of chemical structure on hydrophobic surface of wood (Figure 7). The broad and intense band at 3050–3700 cm^−1^ with its centre at approximately 3334 cm^−1^ corresponded to the O–H stretching vibrations of the surface hydroxyl groups and adsorbed water [29]. A new reflection peak appeared near the 2964 cm^−1^, which was due to the methyl group on the PDMS molecule. It was caused by the C–H stretching vibration with the methylene group [32]. Meanwhile, another new reflection peak appeared near 1257 cm^−1^, which was due to the bending vibration reflection peak of Si–O in the PDMS molecule [32]. At the same time, the reflection peak at the wavenumber of 1001 cm^−1^ for the hydrophobic performance of wood significantly enhanced, which was due to the bending vibration reflection peak of Si–O on the surface of the wood after coating of nano and micro hollow mesoporous microspheres [32]. The reflection peak of the modified material at 783 cm^−1^ was significantly reduced, which was due to the elastic vibration reflection peak of Si–C on the wood surface after spraying the microspheres layer [33]. In the untreated wood sample, the peaks at 3334 cm^−1^ and 1001 cm^−1^ were particularly obvious, but after spraying the resin base coating, it could be seen that the O–H absorption peak was reduced with the reflection after the nano and micro hollow mesoporous microspheres spraying for hydrophobic structure coating. The disappearance of the peak indicated that the hydrophobic coating had a modification effect on the wood and could increase the wood hydrophobicity to certain degrees. The nano and micro hollow mesoporous microspheres layer enhanced the hydrophobicity of the wood. Compared with the data obtained from the CA, the conclusions obtained by infrared spectroscopy could better confirm the conclusions above. From the analysis of the infrared spectrum, it was found that the C-H reflection peaks of methyl and methylene groups, and the vibration reflection peaks of Si–CH_3_, Si–O and Si–C indicated that the wood dimensional stability was improved and the formation of superhydrophobicity was achieved.

**Figure 8:**
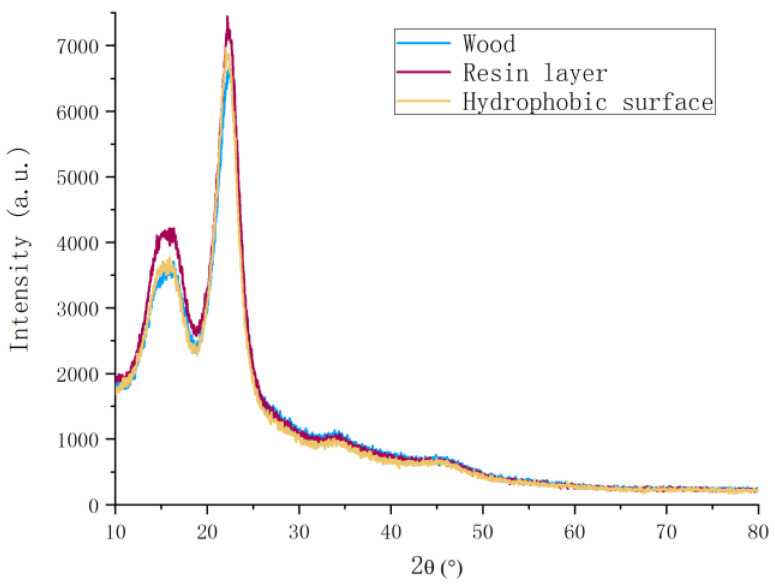
X-ray diffraction pattern of untreated wood samples, resin layer, and hydrophobic surface on wood.

The structure of the wood cell wall was mainly supported by cellulose and filled with lignin and hemicellulose [29]. Cellulose was a two-phase system connected by crystalline and amorphous regions. Its crystalline structure belonged to the monoclinic system. The transition between amorphous regions was gradual, without obvious boundaries, and could be clearly shown by the X-ray diffraction patterns. Figure 8 illustrates the X-ray diffraction patterns of the wood surface with PDMS-based coating of the untreated wood sample, and wood samples with nano and micro hollow mesoporous microspheres coating. It could be seen in Figure 8 that the crystal structure of wood surface with resin layer or nano and micro hollow mesoporous microspheres coating on wood were both very close to the crystal structure of the untreated wood sample. The crystallinity of nano and micro hollow mesoporous microspheres coating was slightly improved. It was revealed that the mixed micro-nano hollow mesoporous microspheres coating could enhance the hydrophobicity of the wood surface and at the same time did not affect the crystal structure of wood or reduce the crystallinity. As well known, the crystallinity of wood directly affects the dimensional stability.

### 3.4. Thermostability of Wood

**Figure 9:**
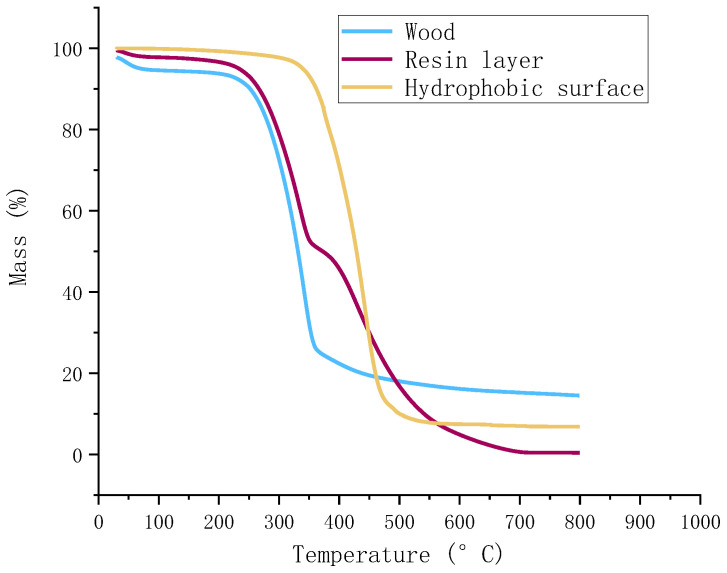
Thermogravimetric curves of untreated wood samples, resin layer, and hydrophobic surface on wood.

The thermal decomposition properties of the untreated wood, resin layer on wood surface, and nano/micro hollow mesoporous microsphere layer with hydrophobic surface presented in Figure 9. As shown in Figure 9, the weight loss of the untreated wood started at around 200 °C, which due to the hemicellulose component of wood began to decompose. At the same time, the rate of weight loss increased rapidly above 200 °C as hemicellulose of the wood decomposed, until approximately 300 °C. It could be seen that the rate of weight loss still increased above 300 °C, reaching its maximum at 350 °C, which was probably because of the decomposition of cellulose and lignin in the wood. The lignin decomposition continued with temperature up to about 800 °C, producing solid carbon [31]. Finally, there was no obvious mass losses shown above 800 °C. With the TG analysis, it was found that the residual mass losses of the untreated wood, resin layer on wood surface, and nano/micro hollow mesoporous microsphere layer with hydrophobic surface were approximately 18.3%, 3.2% and 7.5%, respectively. Meanwhile, the initial weight loss of hydrophobic wood samples was 3.5–4%, as shown in Figure 9, due to the evaporation of free water [34]. Then the initial distinct mass loss of almost 90% occurred in the temperature range of 350–450 °C for the nano/micro hollow mesoporous microsphere layer on wood with the hydrophobic surface. More obvious decompositions took place from approximately 450–600 °C. In general, it was shown that the higher thermal stability was achieved using the nano/micro hollow mesoporous microsphere layer coating on wood surface.

### 3.5. Mechanical Durability of the Hydrophobic Coatings

**Figure 10:**
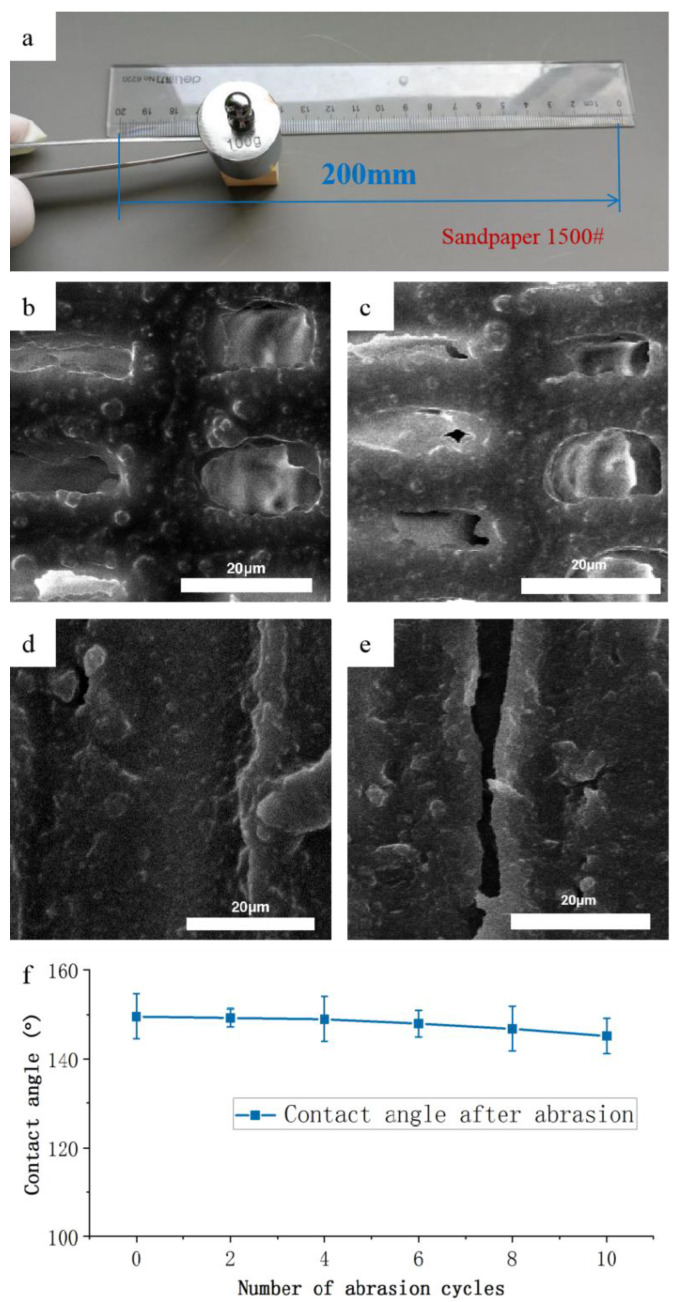
Abrasion resistance test of hydrophobic surface on wood. (**a**) Illustration of the sandpaper abrasion test; (**b**) cross section of wood before abrasion test; (**c**) cross section of wood after abrasion test; (**d**) tangential section of wood before test; (**e**) tangential section of wood after abrasion test; (**f**) the water contact angle as a function of number of abrasion cycles for hydrophobic wood surface.

Hydrophobic coatings were usually mechanically and chemically weak against damages during daily uses [35]. To evaluate the mechanical stability of the hydrophobic wood surface, the friction test was carried out using 1500 mesh sandpaper to examine the abrasion resistance of the hydrophobic wood surface with the best treating conditions that mixed particles concentration, which was 1.0% and spraying 20 times (Figure 10a). Different sections of wood were tested to affirm that the coating on the wood surface had excellent mechanical durability. Before the sandpaper abrasion test, the nano and micro hollow mesoporous microsphere were clearly seen on wood cavity on the cross section (Figure 10b) and the tangential section (Figure 10d) from the examination of the scanning electron microscope. Nevertheless, after the mechanical durability test, the microstructure of the hydrophobic surface of wood was slightly destroyed, but the microsphere coating still could be observed. Therefore, it clearly revealed that the nano and micro hollow mesoporous microsphere-coated wood surface kept its hydrophobic character even after the multiple abrasion tests.

The practical application of hydrophobic wood is mainly concerned with mechanical damage of the vulnerability of the rough surface [35]. Therefore, the scratch test was conducted to evaluate the mechanical durability of the hydrophobic coatings on wood surface. The changes in water CA within 10 times abrasion cycles are presented in Figure 10f. As expected, the water CA of the hydrophobic surface remained around 148–149° in good condition after being scratched repeatedly. This indicated the mechanical stability of the hydrophobic wood surface, which was attributed to the close connection of the nano and micro hollow mesoporous microsphere and resin layer on the wood surface.

## 4. Conclusions

The hydrophobic surface of wood with conspicuous water resistance and persuasive mechanical stability was successfully developed by coating the mixed nano and micron hollow mesoporous microsphere layer. The mixed nano and micron hollow mesoporous microsphere layers exhibited remarkable hydrophobic performance on both the cross and tangential sections of wood, presenting the water contact angle of up to 150°, which can be considered as superhydrophobic performance. Water droplet test manifested a perfect spherical shape of water on the modified wood surface, demonstrating apparently hydrophobic properties. In terms of the results obtained from the abrasion test, the hydrophobic wood surface showed crucial hydrophobic properties after the sandpaper abrasion test and thermostability test, demonstrating a great potential for wide applications in the future industry.

## References

[1] Mi R., Chen C., Keplinger T., Pei Y., He S., Liu D., Li J., Dai J., Hitz E., Yang B. (2020). Scalable aesthetic transparent wood for energy efficient buildings. Nat. Commun..

[2] Ab Latib H., Liat L.C., Ratnasingam J., Law E.L., Azim A.A.A., Mariapan M., Natkuncaran J. (2020). Suitability of Paulownia Wood from Malaysia for Furniture Application. Bioresources.

[3] Zhu H., Luo W., Ciesielski P.N., Fang Z., Zhu J.Y., Henriksson G., Himmel M.E., Hu L. (2016). Wood-Derived Materials for Green Electronics, Biological Devices, and Energy Applications. Chem. Rev..

[4] Amidon T.E., Wood C.D., Shupe A.M., Wang Y., Graves M., Liu S. (2008). Biorefinery: Conversion of woody biomass to chemicals, energy and materials. J. Biobased Mater. Bioenergy.

[5] Laurichesse S., Averous L. (2014). Chemical modification of lignins: Towards biobased polymers. Prog. Polym. Sci..

[6] Esteves B.M., Pereira H.M. (2009). Wood Modification by Heat Treatment: A Review. Bioresources.

[7] Yang T., Ma E., Cao J. (2020). Dynamic moisture sorption and dimensional stability of furfurylated wood with low lignin content. Holzforschung.

[8] Jiang J., Chen Y., Cao J., Mei C. (2020). Improved Hydrophobicity and Dimensional Stability of Wood Treated with Paraffin/Acrylate Compound Emulsion through Response Surface Methodology Optimization. Polymers.

[9] Chen J., Wang Y., Cao J., Wang W. (2020). Improved Water Repellency and Dimensional Stability of Wood via Impregnation with an Epoxidized Linseed Oil and Carnauba Wax Complex Emulsion. Forests.

[10] Yang H., Wang S., Wang X., Chao W., Wang N., Ding X., Liu F., Yu Q., Yang T., Yang Z. (2020). Wood-based composite phase change materials with self-cleaning superhydrophobic surface for thermal energy storage. Appl. Energy.

[11] Li Y., Chen C., Song J., Yang C., Kuang Y., Vellore A., Hitz E., Zhu M., Jiang F., Yao Y. (2020). Strong and Superhydrophobic Wood with Aligned Cellulose Nanofibers as a Waterproof Structural Material. Chin. J. Chem..

[12] Dong S., Hu P., Li X., Hong C., Zhang X., Han J. (2020). NiCo_2_S_4_ nanosheets on 3D wood-derived carbon for microwave absorption. Chem. Eng. J..

[13] Wang L., Li N., Zhao T., Li B., Ji Y. (2019). Magnetic Properties of FeNi₃ Nanoparticle Modified Pinus radiata Wood Nanocomposites. Polymers.

[14] Chen Y., Zhang L., Mei C., Li Y., Duan G., Agarwal S., Greiner A., Ma C., Jiang S. (2020). Wood-Inspired Anisotropic Cellulose Nanofibril Composite Sponges for Multifunctional Applications. ACS Appl. Mater. Interfaces.

[15] Sun L., Xie Y., Ou R., Guo C., Hao X., Wu Q., Wang Q. (2020). The influence of double-layered distribution of fire retardants on the fire retardancy and mechanical properties of wood fiber polypropylene composites. Constr. Build. Mater..

[16] Gibier M., Lacoste C., Corn S., Pucci M.F., Quoc Khoi T., Haurie L., Sonnier R. (2020). Flame retardancy of wood-plastic composites by radiation-curing phosphorus-containing resins. Radiat. Phys. Chem..

[17] Lu X., Hu Y. (2016). Layer-by-layer Deposition of TiO_2_ Nanoparticles in the Wood Surface and its Superhydrophobic Performance. Bioresources.

[18] Dong H., Strawhecker K.E., Snyder J.F., Orlicki J.A., Reiner R.S., Rudie A.W. (2012). Cellulose nanocrystals as a reinforcing material for electrospun poly(methyl methacrylate) fibers: Formation, properties and nanomechanical characterization. Carbohydr. Polym..

[19] Lu Z., Eadula S., Zheng Z., Xu K., Grozdits G., Lvov Y. (2007). Layer-by-layer nanoparticle coatings on lignocellulose wood microfibers. Colloids Surf. A Physicochem. Eng. Asp..

[20] Donath S., Militz H., Mai C. (2004). Wood modification with alkoxysilanes. Wood Sci. Technol..

[21] Okada K., Isobe T., Katsumata K.-I., Kameshima Y., Nakajima A., MacKenzie K.J.D. (2011). Porous ceramics mimicking nature-preparation and properties of microstructures with unidirectionally oriented pores. Sci. Technol. Adv. Mater..

[22] Tshabalala M.A., Sung L.-P. (2007). Wood surface modification by in-situ sol-gel deposition of hybrid inorganic-organic thin films. J. Coat. Technol. Res..

[23] Jamali A., Evans P.D. (2011). Etching of wood surfaces by glow discharge plasma. Wood Sci. Technol..

[24] Janin A., Zaviska F., Drogui P., Blais J.-F., Mercier G. (2009). Selective recovery of metals in leachate from chromated copper arsenate treated wastes using electrochemical technology and chemical precipitation. Hydrometallurgy.

[25] Chereddy S., Aguirre J., Dikin D., Wunder S.L., Chinnam P.R. (2020). Gel Electrolyte Comprising Solvate Ionic Liquid and Methyl Cellulose. ACS Appl. Energy Mater..

[26] Zhao Z., Sakai S., Wu D., Chen Z., Zhu N., Huang C., Sun S., Zhang M., Umemura K., Yong Q. (2019). Further Exploration of Sucrose-Citric Acid Adhesive: Investigation of Optimal Hot-Pressing Conditions for Plywood and Curing Behavior. Polymers.

[27] Tan Y., Wang K.L., Dong Y.M., Zhang W., Zhang S.F., Li J.Z. (2020). Bulk superhydrophobility of wood via in-situ deposition of ZnO rods in wood structure. Surf. Coat. Technol..

[28] Tu K., Kong L., Wang X., Liu J. (2016). Semitransparent, durable superhydrophobic polydimethylsiloxane/SiO_2_ nanocomposite coatings on varnished wood. Holzforschung.

[29] Zhao Z., Sakai S., Wu D., Chen Z., Zhu N., Gui C., Zhang M., Umemura K., Yong Q. (2020). Investigation of Synthesis Mechanism, Optimal Hot-Pressing Conditions, and Curing Behavior of Sucrose and Ammonium Dihydrogen Phosphate Adhesive. Polymers.

[30] Segal L., Creely J.J., Martin A.E., Conrad C.M. (1959). An Empirical Method for Estimating the Degree of Crystallinity of Native Cellulose Using the X-ray Diffractometer. Text. Res. J..

[31] Yang R., Liang Y., Hong S., Zuo S., Wu Y., Shi J., Cai L., Li J., Mao H., Ge S. (2020). Novel Low-Temperature Chemical Vapor Deposition of Hydrothermal Delignified Wood for Hydrophobic Property. Polymers.

[32] Jia C., Zhang Y., Cui J., Gan L. (2019). The Antibacterial Properties and Safety of a Nanoparticle-Coated Parquet Floor. Coatings.

[33] Yang R., Cao Q., Liang Y., Hong S., Xia C., Wu Y., Li J., Cai L., Sonne C., Le Quyet V. (2020). High capacity oil absorbent wood prepared through eco-friendly deep eutectic solvent delignification. Chem. Eng. J..

[34] Thi Tham N., Thi Vinh Khanh N., Xiao Z., Wang F., Zheng Z., Che W., Xie Y. (2019). Combustion behavior of poplar (Populus adenopoda Maxim.) and radiata pine (Pinus radiata Don.) treated with a combination of styrene-acrylic copolymer and sodium silicate. Eur. J. Wood Wood Prod..

[35] Zhu Q., Chu Y., Wang Z., Chen N., Lin L., Liu F., Pan Q. (2013). Robust superhydrophobic polyurethane sponge as a highly reusable oil-absorption material. J. Mater. Chem. A.

